# Theoretical Insights into Twist–Bend Nematic Liquid Crystals: Infrared Spectra Analysis of Naphthalene-Based Dimers

**DOI:** 10.3390/ma18091971

**Published:** 2025-04-26

**Authors:** Barbara Loska, Yuki Arakawa, Katarzyna Merkel

**Affiliations:** 1 Institute of Materials Engineering, Faculty of Science and Technology, University of Silesia in Katowice, ul. 75 Pułku Piechoty 1A, 41-500 Chorzów, Poland; barbara.loska@us.edu.pl; 2 Department of Applied Chemistry and Life Science, Graduate School of Engineering, Toyohashi University of Technology, 1-1 Hibarigaoka, Tempaku-cho, Toyohashi 441-8580, Aichi, Japan; arakawa@tut.jp

**Keywords:** FTIR spectroscopy, DFT simulations, conformational probability, liquid crystal dimers, spatially modulated phases, twist–bend nematics

## Abstract

In this study, we employed density functional theory (DFT), a standard method in quantum chemistry, to investigate the structural intricacies of thioether-linked naphthalene-based liquid-crystal dimers. The theoretical analysis included the calculation of the molecular bend angle, a crucial factor influencing the formation of the twist–bend nematic (N_TB_) phase, as well as other molecular parameters such as transition dipole moments, bond lengths, and bond energies. These calculations allowed for the determination of the probable conformations and the computation of their vibrational spectra, which are essential for interpreting experimental spectra. Connecting these insights, we identified stable conformations and observed differences in the spectra between the conventional nematic (N) and N_TB_ phases. The combined DFT calculations and infrared absorbance measurements allowed us to investigate the structure and intermolecular interactions of molecules in the N and N_TB_ phases of the dimers. Notably, significant changes in average absorbance were detected in the experimental spectra in the N_TB_ phase. During the transition from the N phase to the N_TB_ phase, a clear decrease in absorbance for longitudinal dipoles and an increase for transverse dipoles were observed. This phenomenon suggests that longitudinal dipoles are antiparallel, while transverse dipoles are parallel. To verify the influence of nearest-neighbor interactions, DFT calculations were conducted on a system comprising several neighboring molecules.

## 1. Introduction

Liquid crystals occupy a unique realm between isotropic liquids and crystalline solids, exhibiting remarkable properties that have garnered significant interest across various scientific disciplines. Among them, a twist–bend nematic (N_TB_) phase has recently emerged as an intriguing class. The discovery of the N_TB_ phase involved a series of significant contributions. The concept of spontaneous symmetry breaking in mesophases of achiral bent-shaped molecules was initially explored by Meyer in 1973 [1]. This theoretical groundwork laid the foundation for further investigations, which would come a couple of decades later. In 2001, Dozov examined spontaneous symmetry breaking in mesophases of achiral bent-shaped molecules and provided insights into the potential existence of novel liquid-crystal phases, characterized by a negative bend elastic constant [2]. In subsequent investigations, Memmer conducted computer simulation studies in 2002 to explore the lower-symmetry liquid-crystal phases Dozov anticipated, further supporting the existence of these distinct mesophases and providing valuable insights into their characteristics [3].

It took nearly another decade for the experimental confirmation of this intriguing phase to take place. In 2010, Panov et al. provided compelling experimental evidence for the existence of the N_TB_ phase in nonchiral planar-aligned bimesogens [4]. Their groundbreaking work revealed spontaneous periodic deformations and a transition from a nematic (N) to another N phase accompanied by a negative elastic constant, thus confirming the presence of the N_TB_ phase and its distinct characteristics. This experimental verification opened up new avenues of exploration and prompted further investigations into the twist–bend phenomenon. Subsequently, in 2011, Cestari et al. conducted a comprehensive study focusing on the liquid-crystal dimer 1″,7″-bis(4-cyanobiphenyl-4′-yl) heptane (CB7CB) [5]. Their investigation shed further light on the N_TB_ phase, providing detailed insights into its phase behavior and properties.

Another decade later, we are continuing the exploration of twist–bend nematogens, actively searching for new liquid-crystal dimers that exhibit this intriguing phase. N_TB_ phases exhibit a modulated N structure, characterized by the presence of helical arrangements on a nanoscale, with a pitch length spanning a few nanometers [6,7,8]. These helical structures give rise to fascinating optical properties and have drawn considerable attention for their potential applications in advanced display technologies and photonic devices [9,10,11].

While the N_TB_ assignment has been widely adopted to describe the lower-temperature-modulated nematic phase found in bent-shaped dimers such as CB7CB, it is worth noting that this interpretation remains a subject of ongoing debate. In particular, Vanakaras and co-authors have highlighted that the observed nanoscale pitch modulation may not correspond to the twist–bend structure originally envisioned by Meyer [12]. Instead, it has been argued that the observed features of the so-called N_X_ phase are more consistent with a polar-twisted nematic (N_PT_) structure, as described in the theoretical model proposed by Vanakaras and Photinos [13,14,15,16]. While many studies—including the present one—follow the widely accepted N_TB_ nomenclature for clarity and consistency with the earlier literature, the nature of this modulated nematic phase is still under discussion.

In this research, through computational techniques employing density functional theory (DFT), we aim to gain insights into the electronic structure, molecular configurations, and vibrational properties of these intriguing materials. Understanding the fundamental aspects of twist–bend nematics is vital for tailoring their properties and designing novel liquid-crystal materials with enhanced performance. It is widely acknowledged that the formation of the N_TB_ phase necessitates a molecular bending. Therefore, the assessment of conformational probabilities in N_TB_-forming molecules has emerged as an indispensable approach [17,18,19,20]. This is because a singular conformation at the energy minimum is insufficient for comprehensively evaluating molecular curvature and other attributes in flexible liquid-crystal dimers.

Through this paper, we contribute to the broader understanding of twist–bend nematics and advance the knowledge of liquid-crystal materials, opening new avenues for their application in various technological fields. In this study, we present a theoretical and spectroscopic investigation of thioether-linked 6-(4-cyanophenyl)-2-naphtalene-based dimers with the heptyl and the propyl chain in the spacer. The spacer contains an odd number of methylene units, similarly to cyanobiphenyl-based dimers like the archetypal N_TB_-forming CB7CB and its homologs, which have been extensively studied [5,6,8,21,22,23,24,25,26]. Thioether-linked CBS*n*SCB dimers exhibited a smaller dimer bend angle compared to methylene- and ether- linked molecules, facilitating a shorter pitch length of the N_TB_-phase helical formation. Consequently, the dimers examined in this study, denoted as (CN)PNS*n*SNP(CN), feature a thioether linkage; however, in the biphenyl mesogen, the inner phenyl was exchanged for naphthalene. A related molecular design incorporating a naphthalene core has also been reported in the form of benzoyloxynaphthyl-based dimers, which showed comparable liquid crystalline behavior [27]. By employing the polarized infrared (IR) absorbance method on a homogeneously aligned sample, we obtained valuable insights into the orientation of the molecules’ transition dipole moments. These findings were then compared with the theoretically calculated Cartesian components of the vibrational transition dipole moment for specific vibrations, thereby facilitating precise band assignment in the experimental spectra, which allowed for the analysis of the IR spectroscopy measurements.

## 2. Materials and Methods

### 2.1. Materials

**Figure 1:**
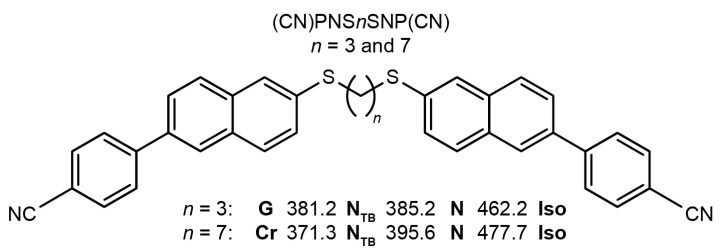
Chemical structure of the studied dimers and their phase-transition temperatures.

In this research, we focused on the investigation of thioether-linked dimers based on the 6-(4-cyanophenyl)-2-naphthyl- rigid core, with the specific dimer acronym (CN)PNS*n*SNP(CN), where PN denotes a phenyl-naphthalene ring core, (CN) is the cyano group, and *n* is the odd number of carbons in the alkyl spacer. The terminal groups of the dimer are chemically linked to each other through thioether bridges. Thioether-linked dimers, which incorporate asymmetric π-conjugated mesogenic arms with terminal cyano groups, have been shown to offer significant advantages in realizing materials that exhibit a diverse range of N_TB_ phases and glassy N_TB_ states even at room temperature. Synthesis details and a preliminary DSC investigation of the compounds have been published by Arakawa et al. [28]. Figure 1 illustrates the chemical structure and transition temperatures of the studied dimers determined by Arakawa et al. by means of Differential Scanning Calorimetry (DSC).

### 2.2. Polarizing Optical Microscopy

**Figure 2:**
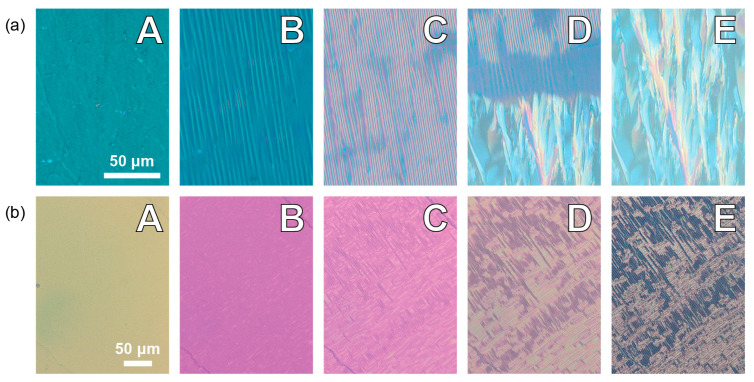
Textures observed during cooling at a rate of 2 K/min: (**a**) (CN)PNS7SNP(CN) sample: A—N phase (445 K); B—N–N_TB_ transition (396 K); C—N_TB_ phase (392 K); D—N_TB_-Cr transition (390 K); E—Cr phase (385 K). (**b**) (CN)PNS3SNP(CN) sample: A—N phase (450 K); B—N–N_TB_ transition (378 K); C—N_TB_ phase (373 K); D and E—N_TB_ glass (363 K and 310 K, respectively). All textures were observed using ZnSe cells with a thickness of 5 µm.

Polarizing Optical Microscopy (POM) was employed to evaluate the alignment quality of the prepared ZnSe cells (Crystan, Dorset, UK), as well as for the preliminary identification of mesophases and determination of phase-transition temperatures. The liquid-crystal textures of the samples were observed using a polarizing microscope (Olympus BX56, Olympus, Tokyo, Japan) equipped with a PID-controlled temperature system, ensuring an accuracy of 2 mK, and are presented in Figure 2.

### 2.3. IR Measurements

The IR measurement cells used in this study achieved planar alignment by sandwiching the dimer material between two optically polished zinc selenide (ZnSe) disks. These disks were spin-coated with an SE-130 commercial polymer aligning agent (Nissan Chemical Industries, Ltd., Pasadena, TX, USA). The aligning agent was rubbed after polymerization and the cell was assembled with an antiparallel rubbing direction to achieve a planar alignment in the sample. The thickness of the fabricated cells was achieved by using a Mylar foil spacer (GoodFellow Ltd., Huntingdon, UK) and determined, by measuring interference fringes using a spectrometer interfaced with a PC (Avaspec-2048, Avantes, Apeldoorn, The Netherlands), to be 5.62 μm and 5.77 μm for the shorter (*n* = 3) and longer (*n* = 7) studied dimers, respectively.

IR spectra were obtained using an Agilent Cary 670 FTIR Fourier IR spectrometer (Agilent Technologies, Santa Clara, CA, USA). The transmission method with a polarized IR beam was employed for the experimental setup, where an IR-KRS5 grid polarizer (Specac Ltd., Orpington, UK) was utilized to polarize the IR beam. The acquired IR spectra were measured across the wavenumber range of 500–4000 cm^−1^. These measurements facilitated the determination of the orientation of transition dipole moments with respect to the long molecular axis and the temperature-dependent behavior of sample absorbance.

Generally, to determine all three components of absorbance (A_x_, A_y_, A_z_), two samples with different orientations are required: planar (homogeneous) and homeotropic. However, achieving a satisfactory homeotropic alignment proved challenging for the tested cyanophenyl-napthalene dimer materials, due to the chemical composition of the terminal groups of the dimer. Consequently, assuming the uniaxial nature of the material, A_x_ was assumed to be equal to A_y_, and the mean absorbance (A_0_) was calculated as A_0_ = (2A_y_ + A_z_)/3. The absorbance components were quantified by measuring the area bound by the contour of a specific band using Bio-Rad Win-IR Pro version 2.96e.

### 2.4. DFT Calculations

Electronic structure calculations of the molecules were performed using the Gaussian16 software package [29]. Density functional theory (DFT) was employed to calculate various molecular properties, including molecular structures, harmonic vibrational force constants, absolute IR intensities, and components of transition dipole moments. The B3LYP functional, which combines Becke’s three-parameter exchange functional with the Lee, Yang, and Parr correlation functional, was utilized. The polarization basis set (6-311G (d,p)) was used for the calculations [30,31].

To calculate the spectral density components, information about the transition dipole moments for specific vibrations was used. The parallel component of the absorption coefficient was determined by squaring the component of the transition dipole moment along the axis coinciding with the long axis of the dimer (|*μ*_z_|^2^ = *μ*^2^_‖_). Similarly, the perpendicular component of the spectral density was determined by summing the squares of the transition dipole moments along the vertical directions (|*μ_x_*|^2^ + |*μ_y_*|^2^ = *μ*^2^_⊥_). The direction of the transition dipole moment was determined according to the molecular reference system.

Theoretical vibrational frequencies were appropriately scaled. According to the Computational Chemistry Comparison and Benchmark Database, for the polarization base and method used, the scaling coefficient was determined to be 0.967 ± 0.021. We found that two scaling factors facilitated better comparability with experimental results, and thus we split the data into two regions: below and above 2000 cm^−1^. We empirically determined the coefficients for these ranges to be 0.9519 and 0.9736, respectively. Both of these values fall within the error margin of the database values. Gaussian profiles with a full width at half maximum (FWHM) of 7 cm^−1^ were employed. The visualization of the results was performed using GaussView 6 [32].

We first calculated the rotational potential barriers of the (CN)PNS3SNP(CN) molecules, using the relaxed potential energy surface scan method with molecular geometry optimization. Relaxed scans about the $\varphi_{1}$ of the molecule angles (36 × 10° steps) while varying the fixed $\varphi_{2}$ angles (18 × 10° steps) were performed. As the energy barrier to the internal rotation in the alkyl chain is very small (approx. 1 kJ/mol), only the all-trans conformation of the linker was considered. Rotational potential barriers were calculated considering the minimum energy of the whole population of dimers to create a 3D energy barrier map. Subsequently, based on the Cartesian coordinates of the lowest-energy geometries, the angle between the two mesogenic units was calculated.

## 3. Results

### 3.1. Energy Barrier Map and Conformers

**Figure 3:**
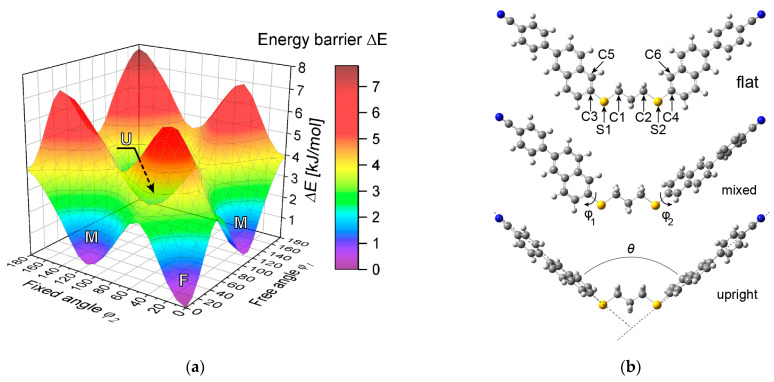
(**a**) A 3D plot of the energy barriers for calculated dimer conformations. M—mixed; F—flat; U—upright conformers. (**b**) The identified minimum-energy conformations: top—the flat conformation with atom number labels; middle—the mixed conformation with torsional angle designations; bottom—the upright conformation and opening angle. Table 1 shows a comparison of the minimum-energy bend angles $\theta_{min}$ and the probability-weighted average bend angle $\theta_{avg}$ for the simulated (CN)PNS3SNP(CN) molecule.

The 3D plot of the rotational potential (energy barrier) map for all considered conformers is presented in Figure 3. The dihedral energy of the absolute minimum among all scans was taken equal to zero. Because of local symmetry, we can identify three distinct minima:

- The symmetrical conformation of minimum energy $\varphi_{1}=\varphi_{2}=0{}^{\circ}$ (referred to as the flat conformation) with energy barrier ΔE= 7.79 kJ/mol;
- The asymmetrical conformation $\varphi_{1}=0$ and $\varphi_{2}=110{}^{\circ}$, with energy barrier ΔE= 7.31 kJ/mol (referred to as the mixed conformation);
- The symmetrical conformation where $\varphi_{1}=\varphi_{2}=110{}^{\circ}$—with an energy barrier of ΔE= 6.19 kJ/mol (referred to as the upright conformation).

For further calculations, only the dimers corresponding to the lowest-energy conformations were investigated. Given the small number of alkyl groups in the linker, only the *all-trans* conformation was considered. Based on the Cartesian coordinates of the atoms of the calculated conformers, we found the bend angles of the molecules and calculated the Boltzmann distribution to obtain the probability-weighted average bend angles of the molecules.

Through the use of the energies of the calculated conformers, the probability-weighted average opening angle $\theta_{avg}$ was calculated to be 100.8°. The introduction of the weighted average based on the Boltzmann distribution to the bend angle calculation did not yield substantial variations in the resulting bend angle values; the observed variance remained within the range of 1.3°. Similar calculations were performed for dimers such as CB9CB, CBS7SCB, and CBS7OCB. The obtained results include the minimum and average values of the bend angles: CB9CB ($\theta_{min}=$ 111.4; $\theta_{avg}=$ 103.7), CBS7SCB $(\theta_{min}=$ 94.1; $\theta_{avg}=$ 79.1), and CBS7OCB ($\theta_{min}=$127.4; $\theta_{avg}=$114.1). In these calculations, the conformations of alkyl chains in the spacer were also taken into account [17,18,19,20].

It is important to note that our calculations did not account for the presence of hairpin conformers. In a real sample, these conformers might lower the average molecular bend. However, determining bend angles for isolated molecules is insufficient for a direct study of the conformational population.

### 3.2. Vibrational Spectra Band Assignments

Achieving perfect experimental order is inherently challenging, and a multitude of factors, particularly intermolecular interactions, influence the dichroism of spectral bands. Consequently, solely relying on the analysis of experimental spectra makes it difficult to definitively pinpoint which band and which para-axis of the dimer components correspond to the behavior of the dimer’s long axis.

For symmetric dimers like the ones studied, each band corresponding to the vibration of the phenyl-naphthalene rigid core is anticipated to yield two characteristic vibrations with similar frequencies but differing intensities. These vibrations, occurring in both arms and characterized by one being in phase while the other is out of phase, can interact and result in a transition dipole moment either along or across the dimer’s long axis. An analysis of band dichroism in the theoretical spectra, representing an ideal order, suggests that if “infinite dichroism” is attained—where one component reaches maximum intensity while the other nears zero—such a band describes the behavior of the dimer’s long axis. Conversely, when band dichroism falls in the intermediate range, indicating a lack of vibration coupling in both arms, this band characterizes the behavior of the mesogens’ para-axes.

Generally, the spectra can be divided into frequency ranges, each associated with specific vibrational characteristics:

- 500–950 cm^−1^—these encompass deformational vibrations involving the carbon atoms (C-C) and involving linker sulfur atoms (C-S-C), as well as deformations of the hydrogen atoms (C-H) oriented out of the plane of the rigid cores of the dimer;
- 1000–1650 cm^−1^—this range encompasses a broad spectrum of vibrational modes, including characteristic deformations within the naphthalene and benzene plane and deformations of the methylene groups within the alkyl chain linker of the dimer;
- 2100–2400 cm^−1^—this range notably features the stretching vibrations of the cyan group (C-N), manifested as a sharp and highly intense peak in the experimental spectrum;
- 2900–3100 cm^−1^—encompassing stretching vibrations of hydrogen atoms (C-H) within the aromatic ring, this range exhibits challenges in theoretical reproduction. The spectral bands in this range pertain to mixed vibrations characterized by significant overlap and distortions, primarily influenced by the Fermi resonance effect.

Table 2 provides a summary of the primary experimental bands observed, along with their assignments to specific vibrational modes of the functional groups. A detailed analysis of the polarized spectra, transition dipole moments, and their comparison with the theoretical spectrum for the most energetically stable conformation was conducted for the molecule with a seven-membered chain and is provided in Table S1 in the Supplementary Materials.

**Figure 4:**
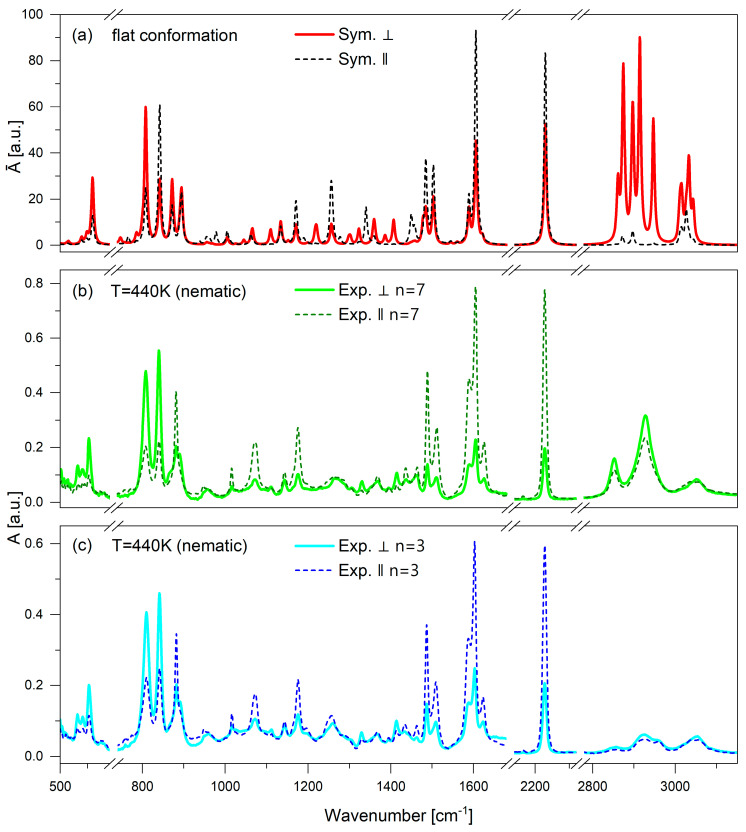
Comparison of the theoretical spectra (B3-LYP/6-311G (d,p)) with the polarized experimental spectra for the (CN)PNS*n*SNP(CN) (*n* = 3, 7) dimers in the region 500–3250 cm^−1^. ‖—parallel absorbance component (in the *z*-axis of the molecular system/sample ordering axis); ⟂—perpendicular absorbance component (sample rubbing direction). (**a**) Theoretical spectra of the (CN)PNS7SNP(CN) molecule, (**b**) experimental spectra of the (CN)PNS7SNP(CN) dimer in the N phase (440 K), and (**c**) experimental spectra of the (CN)PNS3SNP(CN) dimer in the N phase (440 K) (spectra are represented for 5 µm cell).

Figure 4 shows a comparison of the simulated (*n* = 7) and experimental (*n* = 3, 7) spectra. We have previously shown, for similar N_TB_ dimers, that theoretical calculations of the IR spectra for an isolated molecule are better in N compared to N_TB_ experimental results; thus, the experimental results were captured from the N phase (440 K for both materials) [33]. The presented spectra were recorded as a function of incident radiation, where ‖ represents the parallel absorbance component along the *z*-axis of the molecular system or the sample ordering axis, while ⟂ denotes the perpendicular absorbance component corresponding to the sample rubbing direction.

The experimental spectra for the dimers of different spacer lengths reveal a general uniformity for the majority of the vibrational bands. However, a prominent distinction is observed in the bands in 2800–3000 cm^−1^, primarily associated with the stretching vibrations of the methylene (CH_2_) groups. The shorter dimer displays reduced intensity in these bands, which can be attributed to the fewer CH_2_ groups within its spacer. Compared to the theoretical spectra, both experiments show vibrational band overlap. Similarly, differences occurring within the spectral ranges characteristic of the vibrations of CH_2_ groups in alkyl chains are also observed within the range of 1200–1300 cm^−1^, corresponding to the $\gamma_{s}$ and $\gamma_{as}$ bending vibrations of CH_2_.

Another noteworthy distinction between the two dimer lengths pertains to the dichroism of the band at 1600 cm^−1^, defined as the ratio of the parallel to the perpendicular component. The dichroism of the shorter dimer (*R*_3_ = 2.44) aligns better with the simulation (*R_theor_* = 2.06), whereas the longer dimer exhibits reduced intensity in the perpendicular band with a dichroism of *R*_7_ = 3.44. The bands at 1590 and 1600 cm^−1^ correspond to vibrations associated with mesogenic units. The former is primarily linked to naphthalene vibrations, while the latter involves benzene vibrations, with the transition dipole moment parallel to the long molecular axis. Compared to the simulation, there are notable differences in the distribution of intensity and absorbance. The higher-wavenumber shoulder appears more distinct, while the two lower bands coincide to a greater extent in the experimental results.

### 3.3. Simulated Spectra for a Hairpin Conformer

**Figure 5:**
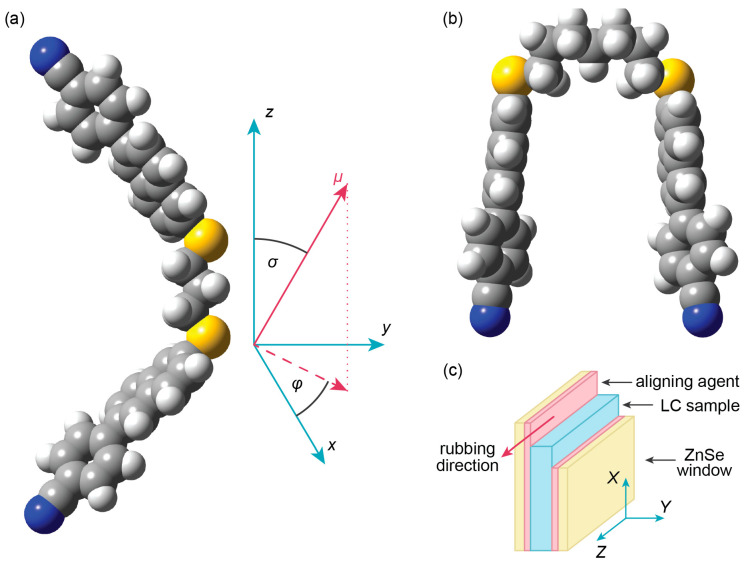
(**a**) Molecular frame of reference: *z*—long axis (bowstring); *x*—normal axis to the bent plane; *y*—bow arrow axis; *σ*—polar angle (between transition dipole *μ* and the *z*-axis of the molecule); *φ* is the azimuthal angle that the transition dipole makes with the *x–z* plane. (**b**) Hairpin conformer. (**c**) The orientation of the laboratory frame (*X, Y, Z*) for the planar sample. In the nematic phase, *Z* was an axis along and *Y* was an axis perpendicular to the optical axis (the optical axis coincided with the rubbing direction). In the N_TB_ phase, *Z* coincided with the helix axis, which was the symmetry element of the N_TB_ phase.

To assess the influence of extreme conformers on the experimental (CN)PNS7SNP(CN) spectrum in the high-temperature N phase (above 400 K), additional DFT calculations were conducted to determine the geometry and vibrational frequencies of more bent conformers. A single (CN)PNS7SNP(CN) molecule was arbitrarily bent into the so-called hairpin conformation, as shown in Figure 5, and optimized.

**Figure 6:**
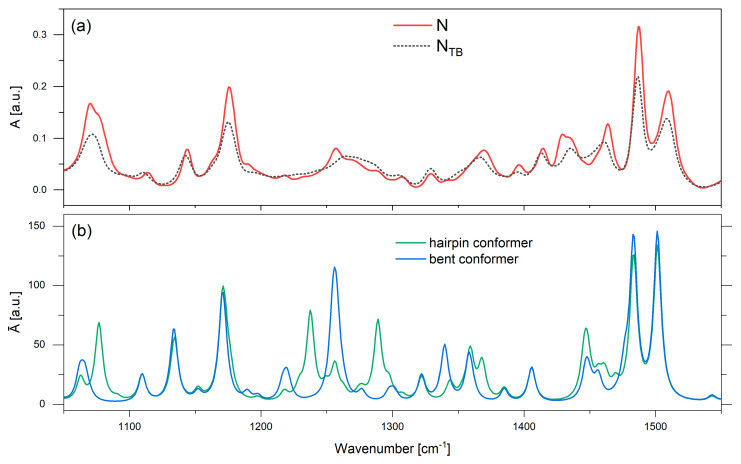
IR spectra of the (CN)PNS7SNP(CN) dimer in the wavenumber range of 1050–1550 cm^−1^. (**a**) Experimental spectra: red solid line—the N phase; black dotted line—the N_TB_ phase. (**b**) Theoretical spectra (B3-LYP/6-311G (d,p)): green line—the hairpin; blue line—the bent conformers.

It is worth noting that in this case, the molecular reference system *z*-axis does not represent the long molecular axis, which, for the hairpin conformer, would be along the mesogen axis (see Figure 5). For this reason, the comparison of the polarized spectra for hairpin and bent conformers would be very difficult, since the distribution between the parallel and perpendicular transition dipole moment contributions for each of the bands is distorted (see Figure S1). It is more informative to compare unpolarized theoretical spectra for the bent and hairpin conformers vs. the experimental spectra in the N and N_TB_ phases (Figure 6).

The main differences between the two calculated conformers occurred in three areas: the range from 1050 to 1100 cm^−1^, that from 1200 to 1375 cm^−1^, and around 1450 cm^−1^. Comparing the theoretical calculations with the experiment, one could conclude that the hairpin conformation bands which are not present in the bent conformation spectrum correlate with the experimental spectrum in the N phase. These hairpin bands are associated with the following linker vibrations:

- In-plane bending vibrations of the CH_2_ groups (rocking $\delta_{s}^{-}$ CH_2_) at 1076 cm^−1^;
- Out-of-plane bending vibrations of the CH_2_ groups (wagging γ_s_ CH_2_) at 1237 cm^−1^ and at 1367 cm^−1^;
- Out-of-plane bending vibrations of the CH_2_ groups (twisting γ_as_ CH_2_) at 1289 cm^−1^;
- In-plane bending vibrations of the CH_2_ groups (scissoring β_s_ CH_2_) at 1447 cm^−1^.

There is only a significant contribution from linker vibration bands.

### 3.4. Experimental Results—Temperature Dependencies of Absorbance

**Figure 7:**
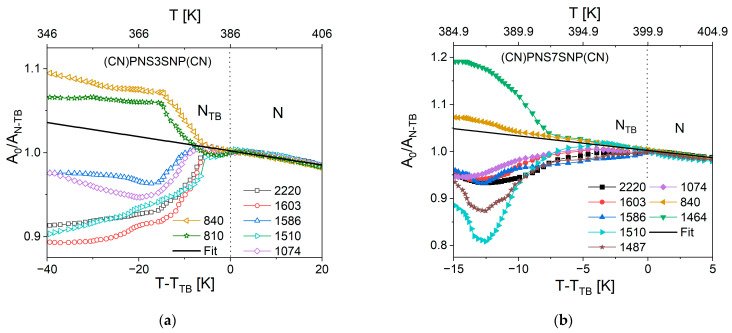
Normalized IR absorbance vs. temperature behavior of the studied dimers: (**a**) for *n* = 3 (open symbols) and (**b**) for *n* = 7 (solid symbols). Longitudinal dipole moment bands: □/■—2200 cm^−1^ (νCN); ○/●—1603 cm^−1^ (br Ph); △/▲—1586 cm^−1^ (br Nap); ▷/▶—1510 cm^−1^ (βCC ip Ph); ★—1487 cm^−1^ (βCC ip Nap +βCH_2_ scissoring); ◇/◆—1074 cm^−1^ (βCC ip Nap + ν CCC). Transversal dipole moment bands: ◁/◀—840 cm^−1^ (γCH op Ph); ☆—810 cm^−1^ (γCH op Nap + C_Ar_S); ▼—1464 cm^−1^ (βCH_2_ scissoring + βCC Nap). Black line: nematic trend extrapolated to the lower temperature N_TB_ phase.

We analyzed the temperature dependence of the average IR absorbance of the materials, defined as the average of each of the directional absorbance components (see Section 2.3), both in the N and N_TB_ phases. Within the N phase, the average absorbance increases with molecular density. However, below the N–N_TB_ transition, the behavior becomes dependent on the orientation of the transition dipole moment corresponding to each vibrational band. To track the reorganization of molecular order during the transition from the nematic to the twist–bend phase, we calculated the mean absorbance for several vibrational bands, each representing a distinct molecular segment. For longitudinal dipoles characterizing the overall molecular axis, we selected bands associated with the spacer (1463 cm^−1^, CH_2_ scissoring) and with naphthalene vibrations involving both the sulfide bridge and the spacer (1074, 1487, and 1586 cm^−1^). Bands reflecting the behavior of the mesogenic arms (and thus the arms of the dimer) include 1603, 1510, and 2200 cm^−1^, attributed to phenyl ring and cyano group vibrations. For these bands associated with the long dimer axis, a distinct decrease in average absorbance is observed at the onset of the N_TB_ phase. In contrast, the bands characterizing only the mesogenic arms show a delayed response—approximately 10 K below the transition temperature. Similar observations were made for the transverse dipole moment bands. Figure 7 presents the normalized mean IR absorbances for transversal and longitudinal transition dipole moments for both of the studied materials.

For transversal dipole moments, the average absorbance trends continue smoothly from the N phase into the N_TB_ phase. Only a slight kink is observed at the transition temperature for both dimers, while at approximately 10 K below the transition temperature, a sudden increase in the average absorbance is observed for all bands. This observation is generally consistent with previous reports on N_TB_-forming dimers, such as those based on cyanobiphenyl cores, where an increase in mean absorbance was noted approximately 30 K below the N–N_TB_ transition [34]. Notably, in the earlier study, dimers with two thioether linkages exhibited a sharp increase at the transition, whereas the (CN)PNS*n*SNP(CN) dimers studied here show a more gradual change. Furthermore, in our case, no significant increase was observed in the lower-N_TB_-temperature region. This is likely due to the rapid vitrification of the samples, which prevented the molecular reorganization necessary for the development of full transversal order, as molecular motion was significantly restricted.

In contrast, for longitudinal dipole moments, the average absorbance generally increases upon cooling through the nematic phase, although this trend slows as the system approaches the N–N_TB_ transition. At the transition point, a distinct drop in absorbance is observed, reflecting a reorganization of the molecular structure. At the same time, the transverse dipole moments continue to follow the nematic trend, showing a further increase in absorbance in the N_TB_ phase. These findings suggest that upon entering the twist–bend phase, longitudinal dipoles tend to adopt an antiparallel arrangement—manifested as a decrease in absorbance—while transverse dipoles align more cooperatively, leading to an increased absorbance. This indicates the onset of bond ordering, where specific orientations of neighboring dipoles become energetically favorable. Such behavior is not observed in the nematic phase, where the absorbance for both dipole types generally increases with cooling due to the rise in molecular density alone, without evidence of directional correlation.

Motivated by these observations, we sought to explore the molecular origin of this dipole reorganization. We hypothesized that the observed bond ordering may arise from short-range, lateral interactions between neighboring molecules, specific to the N_TB_ structure. To test this, we performed DFT simulations incorporating nearest-neighbor molecular arrangements, allowing us to assess how such local interactions can affect the transition dipole moments of individual vibrational bands.

## 4. Discussion

**Figure 8:**
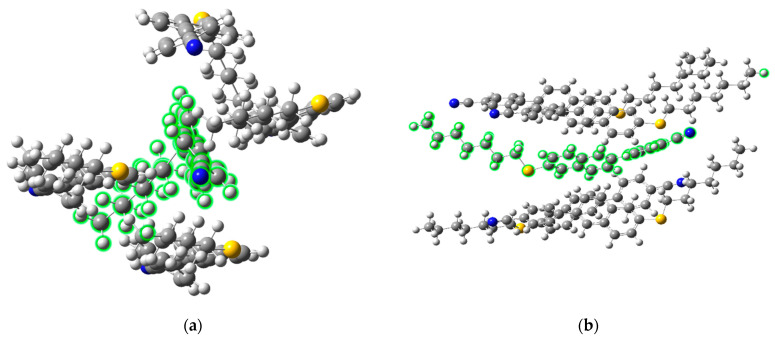
View of the (CN)PNS7 monomer system. (**a**) A top view (X–Y plane) of the system with the arrangement of the five molecules after optimization by the B3LYP/6-311G (d,p) method. The distances between the molecules range from 5 to 6 Å. IR spectra were calculated for a molecule (highlighted in green) that is surrounded by other molecules. (**b**) A side view of the system.

Focusing on the nearest-neighbor interactions and considering the high computational cost of DFT simulations for large molecular systems, we performed calculations for the (CN)PNS7 monomers. In the next step, six monomeric molecules, each in an optimized planar conformation, were arranged into a so-called sublayer (Figure 8). The molecules were placed parallel to each other, with an intermolecular separation of approximately 5 Å, and the cyano groups were arranged in an alternating fashion. This distance was estimated based on experimental data from resonant X-ray scattering (TReXS) studies of the N_TB_ phase for other cyanobiphenyl-based dimers [35]. However, no X-ray data were available for this specific material. The system was optimized using the B3LYP/6-311G (d,p) method.

**Figure 9:**
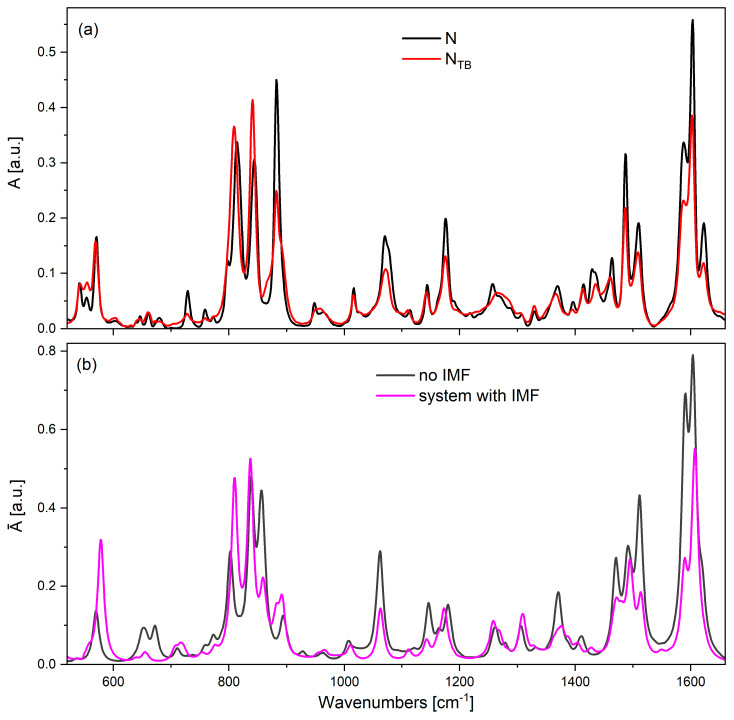
Comparison of the theoretical and experimental spectra for the (CN)PNS7SPN(CB) dimer. (**a**) Experimental spectra: solid black line—the N phase; solid red line—the N_TB_ phase (spectrum is represented for 5 mm cell). (**b**) Theoretical spectra for the (CN)PNS7 monomer: solid gray line—the isolated molecule (no IMF); solid magenta line—a system of five interacting molecules (with IMF).

Following optimization, the outermost molecules within the sublayer were frozen, and vibrational frequencies and intensities were calculated for the central molecule, which was surrounded by the neighboring species (see Figure 8; the selected molecule is marked in green). This methodology enabled the computation of the IR spectrum by determining the transition dipole moments for individual bands while incorporating the influence of the immediate molecular environment. The optimization process revealed nanoscale segregation within the pseudolayers—naphthalene cores preferentially aligned adjacent to one another, forming an aromatic domain, while the flexible spacer adopted a parallel configuration, contributing to the formation of an aliphatic layer. In this arrangement, the naphthalene units of adjacent molecules exhibited partial overlap with the phenyl groups, while the phenyl rings were oriented at an angle with respect to neighboring molecules. A partial overlap of the aromatic moieties and an attraction between the cyano groups and alkyl chains were also observed. The theoretical IR spectrum for the centrally positioned molecule, where intermolecular forces (IMFs) were explicitly considered, was compared with the spectrum of an isolated molecule as well as experimental spectra recorded in the N and N_TB_ phases (Figure 9).

A decrease in intensity was detected for the longitudinal dipole-related bands (wavenumbers: 880, 1060, 1490, 1510, 1600, and 2220 cm^−1^) in the IMF-influenced system compared to an isolated monomer. This trend aligns with the experimental observations, where a decrease in mean absorbance for these bands was noted during the transition from the N to the N_TB_ phase (Figure 4). Conversely, an increase in intensity was noted for the transverse transition dipole moments, specifically at 807 and 850 cm^−1^. This suggests a correlation of transverse dipoles perpendicular to the N order, commonly referred to as bond ordering. The intensity of the 807 cm^−1^ band increased by approximately 67% relative to the isolated molecule, while the 850 cm^−1^ band exhibited a 15% increase. The 807 cm^−1^ band corresponds to out-of-plane CH vibrations of the naphthalene moiety, whereas the 850 cm^−1^ band is associated with out-of-plane CH vibrations of the phenyl ring.

For the bands linked to longitudinal dipoles, a reduction in intensity was observed—approximately 57% for in-plane CC stretching modes of the naphthalene group (1590 cm^−1^, 1060 cm^−1^) and around 15% for characteristic vibrations of the phenyl group (1606 cm^−1^, 1510 cm^−1^). The smallest intensity change was noted for the CN stretching vibration at 2220 cm^−1^ (~10%). These computed values are consistent with experimental trends, where a decrease in the mean absorbance of longitudinal dipole bands (by ~30%) and an increase in transverse dipole bands (by ~25–35%) were recorded upon transitioning from the N to the N_TB_ phase.

It is important to emphasize that interactions involving a limited number of molecules do not fully account for the collective behavior of the system. To achieve a more comprehensive understanding, thermodynamic and statistical considerations—such as entropy and enthalpy contributions—must be incorporated. Therefore, the current calculations should be considered a preliminary step toward more advanced simulations involving the periodic density functional theory, which we are currently trying to carry out.

## 5. Conclusions

It is widely accepted that the molecular curvature of dimers significantly influences the formation and stability of the N_TB_ phase. This study reinforces that view, demonstrating that precise molecular architecture, including both curvature and intermolecular interactions, is critical to realizing materials suitable for technological applications. Our combined theoretical and experimental investigation provides a comprehensive analysis of the structural and spectroscopic properties of naphthalene-based liquid-crystal dimers, offering insights into the mechanisms driving N_TB_ phase behavior.

Through detailed IR spectroscopic analysis, including both experimental and simulated spectra, we successfully assigned characteristic vibrational bands and identified distinct spectral features differentiating the N and N_TB_ phases. Importantly, we showed that the low torsional barrier of the sulfide bridge (~3 kJ/mol) allows neighboring molecules to induce significant conformational changes, influencing the vibrational dynamics of dimers in the N_TB_ phase. Our comparison between discrete low-energy conformers and the Boltzmann-averaged bending angle suggests that while flexibility exists, the average curvature remains relatively stable across conformational populations.

One of the most striking findings of this work is the evolution of dipole correlations during the N-to-N_TB_ phase transition. A pronounced decrease in longitudinal dipole absorbance, accompanied by an increase in transverse dipole absorbance, was observed. This shift indicates a reorganization of molecular alignment, where longitudinal dipoles become negatively correlated due to antiparallel mesogen arrangements, while transverse dipoles align cooperatively. These results point to a highly specific and cooperative reorientation process occurring at the molecular level during N_TB_ phase formation.

To elucidate the molecular basis of this self-organization, we performed DFT modeling incorporating nearest-neighbor interactions. These simulations underscore the crucial role of the local molecular environment in stabilizing the N_TB_ phase. In particular, weak noncovalent interactions—such as π–π stacking between aromatic cores and potential weak hydrogen bonds—emerge as important contributors to phase stability and heliconical ordering.

By integrating experimental IR spectroscopy and quantum chemical modeling, this study offers a multiscale understanding of how molecular structure and interactions govern N_TB_ phase formation in liquid-crystal dimers. These insights advance the fundamental understanding of self-organizing liquid crystalline systems and contribute to the rational design of new materials with tailored mesophases for future applications.

## Figures and Tables

**Table 1:** Energy barrier, ΔE, and molecular bend angles for the global energy minimum conformation, $\theta_{min}$, of the calculated (CN)PNS3SNP(CN) molecule.

Conformation	Torsional Angles	$\mathrm{Potential}\;\mathrm{Energy}\;\left[\frac{\mathrm{kJ}}{\mathrm{mol}}\right]$	$\theta_{min}\left[{}^{\circ}\right]$
Flat	$\varphi_{1}=\varphi_{2}=0{}^{\circ}$	−6,122,426.6725	99.3
Mixed	$\varphi_{1}=0{}^{\circ}$ $\varphi_{2}=110{}^{\circ}$	−6,122,426.1981	99.5
Upright	$\varphi_{1}=\varphi_{2}=110{}^{\circ}$	−6,122,425.1571	100.3

**Table 2:** Vibrational frequencies, IR intensities, and assignments of the most important bands in the (CN)PNS*n*SNP(CN) dimer series.

$\nu \left[cm^{-1}\right]$	$I_{rel}$	Assignment	μ
570	w	γCC op rc + δC_Ar_S + δCN	⊥
810	s	γCH op Nap	⊥
842	s	γCH op Ph	⊥
883	m	βCC ip RC (breathing) + ν_as_ C_Ar_S	||
1016	vw	βCC ip Ph	||
1073	w	βCC ip Nap + ν_Al_ CCC	||
1145	vw	βCH ip Nap	||
1175	w	βCH ip RC	||
1264	vw	βCH ip Nap	||
1463	vw	βCH_2_ (scissoring) + βCC Nap	||
1487	s	βCC Nap + βCH_2_ (scissoring)	||
1510	w	βCC Ph	||
1587	m	br Nap	||
1602	vs	br Ph	||
2223	vs	νCN	||

Key: ip—in-plane vibration; op—out-of-plane deformation; br—stretching and deformation vibrations of the ring (benzene ring); s—symmetrical; as—asymmetrical; Al—alkyl chain; Ar—aromatic ring; Ph—phenyl; Nap—naphthalene; RC—rigid core; ν—stretching; γ—deforming out of plane; β—deforming in plane; δ—deforming; vs—very strong; s—strong; m—medium; w—weak; vw—very weak; *I_rel_*—relative intensity of the bands, $\nu \left[cm^{-1}\right]$—wavenumber.

## Data Availability

The original data presented in the study are openly available in RepOD at https://doi.org/10.18150/IUMWS0 [RepOD, V1]. Data available: raw IR measurement data and DFT simulation data. The date access: 25 April 2025.

## Supplementary Materials

The following supporting information can be downloaded at https://www.mdpi.com/article/10.3390/ma18091971/s1, Figure S1. Comparison of the theoretical spectra (B3-LYP/6-311G (d,p)) for the bent (top) and hairpin (bottom) conformers; Table S1. Theoretical and experimental frequencies, dichroism values, relative intensity, direction of the transition dipole moment, and approximate band assignments for (CN)PNS7SNP(CN).

## Abbreviations

The following abbreviations are used in this manuscript:

N	Conventional Nematic
N_TB_	Twist–Bend Nematic
DFT	Density Functional Theory
DSC	Differential Scanning Calorimetry
IR	Infrared
POM	Polarizing Optical Microscopy
TReXS	Tender Resonant X-ray Scattering
IMFs	Intermolecular Forces
PN	Phenyl-naphthalene Ring Core
CN	Cyano Group
ZnSe	Zinc Selenide
FWHM	Full Width at Half Maximum
CB7CB	1″,7″-Bis(4-cyanobiphenyl-4′-yl) Heptane
(CN)PNS*n*SNP(CN)	6-(4-Cyanophenyl)-2-naphtalene-Based Dimers With the Heptyl and the Propyl Chain in the Spacer
