# Supplementary material for: Theoretical Insights into Twist–Bend Nematic Liquid Crystals: Infrared Spectra Analysis of Naphthalene-Based Dimers
Source: Materials (Basel). 2025 Apr 26;18(9):1971. doi: 10.3390/ma18091971 (PMC12072216; doi:10.3390/ma18091971)
Supplement: Supplementary file 1 [file materials-18-01971-s001.zip › materials-3572659-supplementary.pdf]

materials

Supplementary material

Theoretical Insights into Twist-Bend Nematic Liquid Crystals: Infrared Spectra Analysis of Naphthalene-Based Dimers

Barbara Loska [1], Yuki Arakawa [2] and Katarzyna Merkel [1,*]

[1] Institute of Materials Engineering, Faculty of Science and Technology, University of Silesia in Katowice, ul. 75 Pułku Piechoty 1A, 41-500 Chorzów, Poland; barbara.loska@us.edu.pl

[2] Department of Applied Chemistry and Life Science, Graduate School of Engineering, Toyohashi University of Technology, 1-1 Hibarigaoka, Tempaku-cho, Toyohashi 441-8580, Aichi, Japan; arakawa@tut.jp

* Correspondence: katarzyna.merkel@us.edu.pl

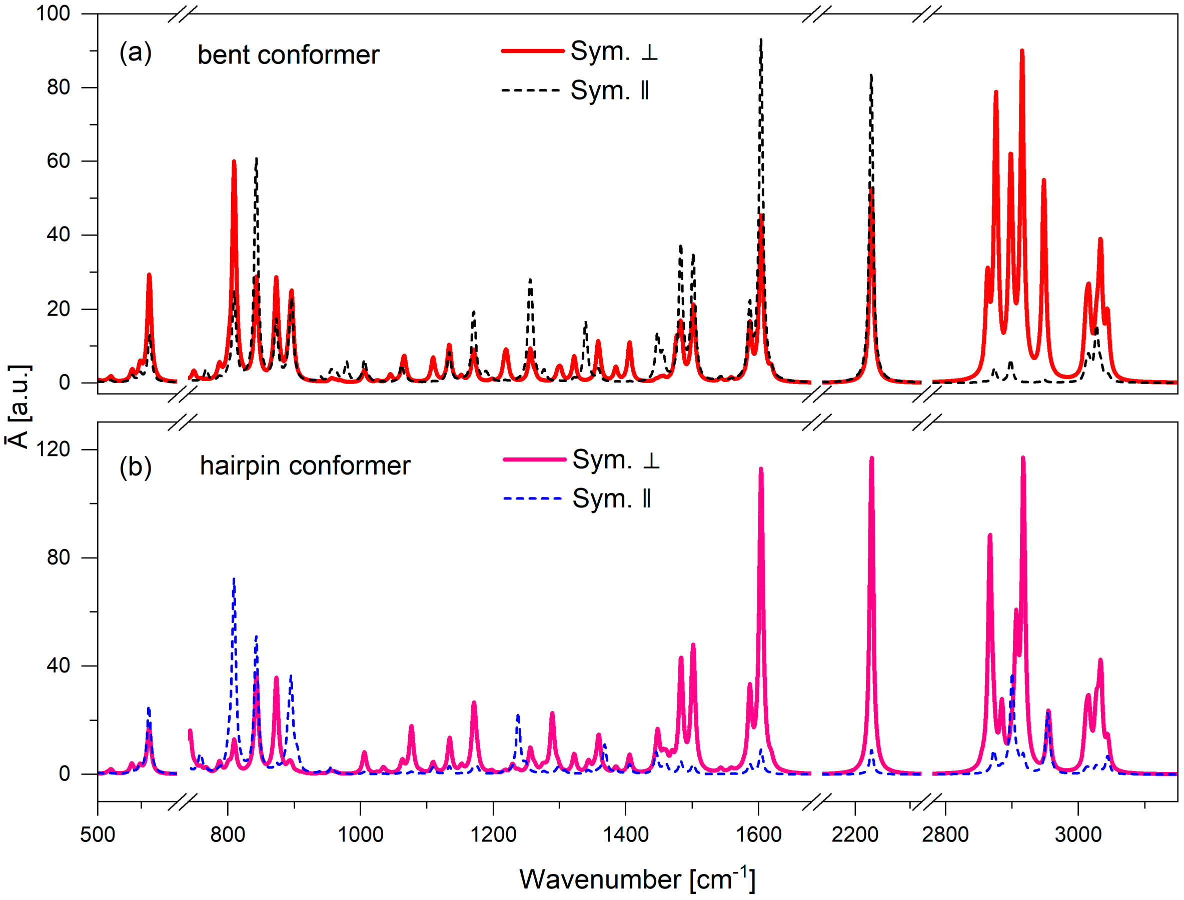

Figure S1 . Comparison of the theoretical spectra (B3-LYP/6-311G (d,p)) for the bent (top) and hairpin (bottom) conformers.

Key for Table S1.

Simulated IR Spectra:

Linear spectra:

ν* – the frequency of normal vibrations [cm^{-1}], I – intensity [Km/mol].

Band spectrum:

ν – vibration frequency expressed in wave numbers [cm-1] scaled with a scaling factor (0.9736 for wavenumbers below 2000 cm^{-1}, 0.9519 for wavenumbers above 2000 cm^{-1}),

I_{rel} – Relative intensity of the bands,

|| or ⊥ – parallel or perpendicular component of spectral density,

R – dichroism $R = \mu_{\parallel}^2/\mu_{\perp}^2$

μ – direction of the transition dipole moment according to the molecular reference system, $\perp_{yz}$ – bend plane of dimer, $\perp_{xz}$ – perpendicular to the bend plane, $||_z$ – long axis of the dimer (bowstring).

Experimental Spectra:

ν – wavenumber [cm-1],

I_{rel} – Relative intensity of the bands,

$A_{\parallel}$– spectrum with polarizer set to ω = 0°,

$A_{\perp}$ – spectrum with polarizer set to ω = 90°,

R – dichroism,

μ – direction of the transition dipole moment.

Key of vibration assignment:

ip – in plane vibration, op – out of plane deformation, br – stretching and deformation vibrations of the ring (benzene ring), s – symmetric, as – asymmetric, Al – alkyl chain,

Ar – aromatic ring, Ph – phenyl, Nap – naphthalene, rc – rigid core (both naphthalene and phenyl ring), ν – stretching, γ – deforming out of plane, β – deforming in plane, δ – deforming, v_s – very strong, s – strong, m – medium, w – weak, vw – very weak, sh – shoulder.

Table S1. Theoretical and experimental frequencies, dichroism values, relative intensity, direction of the transition dipole moment and approximate band assignments for (CN)PNS7SNP(CN).

Simulated							Experimental					Assignment
Linear spectra		Band spectra										
ν^* [cm^{-1}]	I $\left[\frac{Km}{mol}\right]$	ν [cm^{-1}]	I_{rel}		R	μ	ν [cm^{-1}]	I_{rel}		R	μ	
			∥	⊥				∥	⊥			
593.3	29.2	578.3	vw	w	0.46	⊥yz	569.9	vw	m	0.43	⊥	γCC op rc + δC_{Ar}S + δCN
593.9	12.8											
681.8	6.2	664.0	vw	vw	0.71	⊥xz	662.4	vw	vw	0.70	⊥	γCC op rc S + βCSC+ βPh
682.2	4.4											
739.5	4.0	721.4	vw	vw	3.09	ǀǀ z	728.0	vw	vw	1.22	ǀǀ	νC_{Al}S + νCC skeletal
740.8	17.2											
831.2	59.2	809.1	w	s	0.41	⊥yz	810.0	w	vs	0.42	⊥	γCH op Nap
831.3	24.0											
865.8	60.5	843.1	s	w	2.11	ǀǀ z	841.8	w	vs	0.41	⊥	γCH op Ph
865.8	27.7											
896.7	16.1	873.3	vw	w	0.61	⊥yz	-	-	sh	sh	-	βCC ip rc + νC_{Ar}S + breathing Ph
896.8	26.5											
919.9	21.0	895.7	w	w	0.92	⊥yz	883.2	m	w	1.96	ǀǀ	γCH op Nap
920.0	21.6											
1033.1	6.0	1006.7	vw	vw	1.80	ǀǀ z	1016.3	vw	vw	2.13	ǀǀ	βCC ip Ph
1033.1	3.3											
1090.8	4.8	1062.2	vw	vw	1.04	ǀǀ z	1073.2	w	vw	2.62	ǀǀ	βCC ip Nap + γ_{as}CH2 twisting
1091.8	2.0											
1139.7	1.2	1109.9	vw	vw	0.07	⊥xz	1110.8	vw	vw	0.95	⊥	βCH ip Ph
1139.7	5.5											
1164.4	8.1	1134.3	vw	vw	0.80	⊥xz	1143.6	vw	vw	1.34	ǀǀ	βCH ip Nap
1164.5	10.1											
1202.4	16.5	1171.2	w	vw	2.15	ǀǀ z	1176.4	w	vw	2.60	ǀǀ	βCH ip rc
1202.4	8.2											

Table S1. Cont.

1290.1	8.8	1255.9	w	vw	3.00	∥ z	1264.1	vw	vw	1.08	∥	βCH ip Nap
1292.0	14.7											
1358.0	0.7	1322.2	vw	vw	0.21	⊥xz	1329.7	vw	vw	0.57	⊥	βCH ip rc
1358.0	6.9											
1375.9	16.4	1339.7	vw	vw	19.57	∥ z	1329.7	vw	vw	0.57	⊥	$\gamma_s CH_2$ wagging
1394.4	2.8	1358.2	vw	vw	0.36	⊥xz	1367.3	vw	vw	1.24	∥	βCC ip Nap
1394.4	0.2											
1394.9	7.3											
1395.4	3.2											
1443.9	0.3	1405.8	vw	vw	0.04	⊥xz	1413.6	vw	vw	0.60	⊥	βCH ip Ph
1443.9	10.5											
1495.8	6.2	1456.5	vw	vw	4.49	∥ z	1462.8	vw	vw	1.33	∥	βCH ip Nap
1495.8	0.7											
1523.4	36.9	1483.8	m	vw	2.27	∥ z	1486.9	s	w	3.47	∥	νCC + βCC Nap
1523.5	14.2											
1542.1	33.5	1501.3	w	w	1.64	∥ z	1510.0	w	vw	3.09	∥	νCC + βCC Ph
1542.1	20.5											
1629.5	19.0	1587.0	w	vw	1.34	∥ z	1587.1	m	w	3.29	∥	br Nap
1629.6	15.0											
1647.3	93.2	1603.5	vs	m	2.06	∥ z	1602.6	vs	m	3.45	∥	br Ph
1647.4	45.0											
2336.4	84.6	2224.6	vs	m	1.58	∥ z	2223.5	vs	w	3.97	∥	νCN
2336.5	53.4											
3007.5	25.0	2863.3	vw	w	0.02	⊥yz	2854.2	vw	w	0.74	⊥	$\nu_s CH_2$
3020.9	74.8	2875.7	vw	vs	0.04	⊥yz	-	-	sh	sh	-	
3044.3	6.3	2898.5	vw	s	0.10	⊥yz	2930.3	w	m	0.74	⊥	
3044.6	53.8											
3062.4	87.3	2915.6	vw	vs	0.00	⊥xz	-	-	sh	sh	-	$\nu_{as} CH_2$
3096.8	1.1	2948.0	vw	s	0.02	⊥xz	-	-	sh	sh	-	$\nu_{as} CH_2$
3097.0	53.4											
